# Molecular epidemiological characteristics of pertussis in a pediatric hospital in Jinan City, China, 2022 to 2024

**DOI:** 10.3389/fpubh.2025.1657953

**Published:** 2025-09-02

**Authors:** Miao Liu, Xiaoling Wei, Jing Sun, Yanqin Liu, Lu Cheng, Yuling Han, Xiang Ma

**Affiliations:** ^1^Department of Respiratory Diseases, Children's Hospital Affiliated to Shandong University (Jinan Children’s Hospital), Jinan, Shandong, China; ^2^Jinan Key Laboratory of Pediatric Respiratory Diseases, Jinan Children's Hospital, Jinan, Shandong, China; ^3^Jinan Key Laboratory of Children’s Infectious Diseases Precision Diagnosis and Treatment, Children's Hospital Affiliated to Shandong University, Jinan, Shandong, China

**Keywords:** pertussis, children, targeted next-generation sequencing, epidemiological characteristics, respiratory pathogens, co-detection

## Abstract

**Background:**

Pertussis is a highly contagious disease caused by *Bordetella pertussis* (BP) and remains endemic worldwide, with cyclic epidemics occurring every 2–5 years. Non-pharmaceutical interventions (NPIs) implemented during the COVID-19 have altered the epidemiology of respiratory infectious diseases. Pertussis have increased sharply since mid-late 2023, showing a nationwide epidemic. Understanding the epidemiological shifts is crucial for effective prevention. This study analyzed the prevalence and epidemiological characteristics of pertussis among children with respiratory infections from 2022 to 2024.

**Methods:**

We analyzed BP-positive cases identified by targeted sequencing for multiple respiratory pathogens in children aged 0–18 years with respiratory infections treated at our hospital from January 2022 to December 2024. Demographic characteristics, positivity rates, age distribution, and co-infecting pathogens were retrospectively assessed.

**Results:**

A total of 20,059 samples were included, with an overall BP positivity rate of 9.83%. The A2047G macrolide resistance mutation rate was significantly increased from 0.24% in 2022 to 6% in 2023 and 42.86% in 2024, respectively (*χ*^2^ = 500.22, *p* < 0.001). Annual BP positivity rates showed a significant increase from 2022 to 2024 (14.56, 3.26, and 32.24%, respectively; *χ*^2^ = 2698.353, *p* < 0.001). Peak detection periods were July to October in 2022, extended from July through December in 2023, and persisted from January to October in 2024. The proportion of BP-positive cases aged >3–6 years and 6–18 years showed increasing trends (*P*_trend_ < 0.01 and *P*_trend_ < 0.001, respectively). BP co-detection rates with specific pathogens—human rhinovirus, adenovirus, *Haemophilus influenzae*, *Staphylococcus aureus*, and *Mycoplasma pneumoniae* significantly increased (all *P*_trend_ < 0.05), whereas parainfluenza virus co-infections decreased (*P*_trend_ < 0.05). No significant change occurred in *Streptococcus pneumoniae* co-infections (*P*_trend_ > 0.05).

**Discussion:**

This study reveals significant shifts in pediatric pertussis epidemiology during the post-pandemic era, characterized by rising incidence, non-seasonal surges, increased school-aged cases, and heightened co-detection rates. These findings call for enhanced drug-resistant BP surveillance, strengthened vaccination (especially among school-aged children in close contact with infants), improved clinical recognition, and targeted public health interventions to disrupt transmission.

## 1 Introduction

Pertussis, caused by *Bordetella pertussis* (BP), is an extremely contagious acute respiratory infection that is primarily transmitted via respiratory droplets and aerosols, affecting all age groups. It is both a pathogen responsible for acute respiratory infections (ARIs) in children and a leading cause of death among neonates and infants under 3 months of age, due to severe complications such as pneumonia, prolonged apnea and fatal outcomes (1, 2). In the United States (1940–1948), pertussis mortality was nearly three times that of measles, mumps, varicella, rubella, scarlet fever, diphtheria, poliomyelitis, and meningitis combined (3, 4). Its high mortality and morbidity prompted the development of the first generation of whole-cell pertussis (wP) vaccines, which had been available since the 1920s. Since 1974, the global pertussis immunization expansion program has been implemented (5). Following the introduction of wP vaccines in our country since 1978, the annual incidence and mortality rates have declined significantly, from a pre-vaccination level of (100–200)/100,000 to 0.13/100,000 by 2013 (6, 7). However, concerns about reactogenicity of wP vaccines led to a switch to acellular pertussis (aP) vaccines (8). In most high-income countries, the transition from wP to aP vaccines occurred in the early 2000s, whereas our country utilized both formulations from 2006 to 2013 before transitioning exclusively to aP vaccines (9, 10). However, pertussis resurgence has occurred in countries with high aP vaccination coverage over the past 20 years (11).

Surveillance data from 2008 to 2016 indicated that the annual number of pertussis cases in China remained relatively stable, ranging between 1,612 and 6,658. However, a marked increase was observed from 2017 to 2019, with reported cases rising sharply from 10,390 to 30,027 (12). The outbreak of COVID-19 in late 2019 and the implementation of non-pharmaceutical interventions (NPIs) altered the epidemiological patterns of common respiratory infectious diseases (13). Consequently, the number of pertussis cases dropped to 4,475 in 2020 but rebounded to 9,611 in 2021. A further dramatic surge occurred in 2022 and 2023, with cases exceeding 38,000 annually (12). This upward trajectory culminated in 2024 with a staggering 487,700 cases, reaching the highest level in nearly three decades.

The resurgence has prompted extensive discussion regarding the immunology of pertussis (encompassing the duration and mechanisms of both natural infection-induced and vaccine-induced immunity), the capacity of vaccines to prevent transmission and severe disease, and the impact of bacterial evolution on its ability to evade vaccine-mediated immunity (11). Resolving these problems is made complicated by inadequate case detection and the heterogeneity of surveillance diagnostic methodologies. T-NGS (Targeted Next-Generation Sequencing) is a laboratory method that enables rapid identification of diverse predefined microorganisms (bacteria, fungi, viruses, parasites) and simultaneous detection of antimicrobial resistance genes through pathogen-directed sequence enrichment (14). T-NGS demonstrates applicability for pathogen detection across common clinical specimens, including respiratory samples (e.g., bronchoalveolar lavage fluid, sputum), blood, cerebrospinal fluid (CSF), and stool. Critically, it enables the detection of bacteria that are either undetectable by conventional clinical methods or challenging to culture (15). Evidence from the literature indicates that, compared to conventional serological and PCR methods, T-NGS demonstrates superior diagnostic value for pertussis (16). This technology holds significant potential for pathogen identification in infectious diseases, detection of antimicrobial resistance genes, and public health epidemiological pathogen surveillance (17). To address the urgent need for proactive public health measures in response to emerging challenges in pertussis control, this retrospective study employed T-NGS technology to conduct descriptive statistical analysis of pertussis positive data from Children’s Hospital Affiliated to Shandong University for the period 2022–2024. Furthermore, we explored the underlying factors driving these epidemiological shifts, aiming to provide a reference for formulating regional pertussis surveillance strategies and control measures.

## 2 Methods

### 2.1 Study design and setting

As the only tertiary first-class children’s hospital in Shandong Province, the Children’s Hospital Affiliated to Shandong University serves as the regional pediatric referral center for the province and neighboring areas. With an annual outpatient volume exceeding 1.2 million, and more than 60% of patients come from non-local regions, ensuring epidemiological representativeness.

This study retrospectively summarized and analyzed children with pertussis infection among ARIs treated at Children’s Hospital Affiliated to Shandong University between January 2022 and December 2024. The inclusion criteria for subjects were: aged 0–18 years; meeting the diagnostic criteria for ARI according to the 8th edition of *Zhu Fu tang’s Practical Pediatrics*; positive detection of BP by targeted next-generation sequencing (T-NGS). Exclusion criteria included: subsequent positive detection in the same child within 1 week, and children with incomplete information. Subjects were categorized into six age groups: 0–3 months (0–3 M), >3–6 months (>3–6 M), >6–12 months (>6–12 M), >1–3 years (>1–3Y), >3–6 years (>3–6 Y), and >6–18 years (>6–18 Y). This retrospective study was approved by the Ethics Committee of Children’s Hospital Affiliated to Shandong University (Approval No. SDFE-IRB/P-2022019) and was granted a waiver of informed consent.

### 2.2 Sample collection and pathogen detection

Specimens (nasopharyngeal swabs, sputum, bronchoalveolar lavage fluid, or pleural fluid) were collected from all participants upon clinical presentation, transported to KingMed Center for Clinical Laboratory Co., Ltd., Guangzhou, China, and subjected to nucleic acid extraction and following testing. Different specimen types from the same patient were processed and analyzed independently. Total nucleic acid extraction was performed within 24 h of sample collection. Reverse transcription was conducted immediately thereafter using the Meta Pure DNA & RNA Extraction Kit (KS118, Guangzhou King Create Co. Ltd., Guangzhou, China) according to the manufacturer’s instructions. The generated nucleic acid was resolved in DNase- and RNase-Free Water (Cat# 10977023, Invitrogen, Waltham, United States), stored at −80°C, and tested within 7 days. Targeted next-generation sequencing (T-NGS) was employed to detect respiratory pathogens, mainly including Influenza A virus (Flu A), Influenza B virus (Flu B), Human respiratory syncytial virus (RSV), Human rhinovirus (HRV), Human adenovirus (ADV), Human metapneumovirus (hMPV), Human parainfluenza virus (PIV), *Bordetella pertussis*, *Haemophilus influenzae* (HI), *Staphylococcus aureus* (SA), *Mycoplasma pneumoniae* (MP), *Streptococcus pneumoniae* (SP), etc. Specific primers were designed for highly conserved genomic regions. Reference sequence data were primarily sourced from NCBI Ref Seq/NT and refined through the removal of highly similar redundant sequences. The primer design principles were described in a previous study (18). Library preparation was performed using the UP95™ Respiratory Pathogen Microorganisms Multiplex Testing Kit (KS608-50SHXD96, King Create Biotechnology Co., Ltd., Guangzhou, China) following the manufacturer’s instructions. cDNA was synthesized by reverse transcription of the extracted nucleic acid, followed by steps such as target region enrichment, first-round purification, junction ligation, and second-round purification, completed library construction. The constructed libraries were pooled to homogeneous mass. Qualified pooled library was diluted and denatured, of which 500 μL was loaded onto the KM Mini seq Dx-CN Sequencer (KY301, Kingcreate, Guangzhou, China) using a 2 × 150 bp paired-end sequencing protocol, as per the manufacturer’s instructions. The output sequencing data was transferred to fastq format using the bcl2fastq^1^. Subsequently, FastQC^2^ and MultiQC (19) were utilized for assessing overall sequencing quality. The fastp v0.20.1 (20) was then employed for adapter trimming and low-quality read filtration. The selected human reference genome (version hg19) and pathogenic target genome sequences were downloaded from the NCBI GenBank FTP site^3^. Macrolide-resistance in *Bordetella pertussis* was determined by detecting mutations at 23S rRNA gene, any sample with the presence of A2047G was reported as macrolide resistant.

### 2.3 Quality control

Nuclease-free water (Invitrogen, Waltham, MA, United States) was used as a non-template control (NTC) to detect contamination. Generated libraries were quantified using Equalbit DNA HS Assay Kit (Vazyme Biotech, Nanjing, Jiangsu, China) on an Invitrogen Qubit 3.0/4.0 Fluorometer (Thermo Fisher Scientific, Waltham, MA, United States). This ensured that all samples had a library density of at least 0.5 ng/μL, otherwise, the library would be subjected to re-construction. External positive and negative controls were included in each batch’s run to ensure the validity of results. Only report results conforming to quality control standards, samples failing the test must undergo re-testing after troubleshooting, with re-sampling when necessary.

### 2.4 Outcome measures

To minimize selection bias, all pediatric patients with ARIs who tested positive for BP during the study period were included, irrespective of disease severity or treatment outcomes. The raw data underwent processing to remove erroneous entries and address missing values. For patients with multiple positive tests within the same time period, only the first positive result was retained. Results from different sample types (e.g., sputum, BALF) collected from the same patient were analyzed separately (grouped by specimen type). Analyses included calculating the overall BP-positive rate (based on patient infection status), its distribution across different age groups and calendar months, and the co-detection rates, positivity rates were also calculated for each specimen type group and statistically compared between groups.

### 2.5 Statistical analysis

Statistical analysis was performed using IBM SPSS Statistics software (version 23.0). Categorical data are presented as the number of positive results or positive sample rate (%). Comparisons between groups were conducted using the Chi-square (*χ*^2^) test or Fisher’s exact test, as appropriate. Pairwise comparison between groups was performed using the multiple comparison method, and the test level *α* was adjusted according to the Bonferroni method. The analysis of the changing trend of the pathogen detection rate with age was carried out using the *χ*^2^ trend test (Chi-Square Trend Test). All tests were two-tailed with *α* = 0.05, and *p* value < 0.05 was considered statistically significant.

## 3 Results

### 3.1 General characteristics of children with BP infection

A total of 20,059 ARI samples from children aged 1 h to 17 years were included during 2022–2024, BP was detected in 1,972 (9.83%) samples. The BP positivity rate in 2024 (32.24%, 1,113/3,452) was significantly higher than in 2022 (14.56%, 409/2,810) and 2023 (3.26%, 450/13,797) (*χ*^2^ = 2698.35, *p* < 0.001). Among BP-positive samples, the detection rate of the A2047G mutation surged to 42.86% in 2024, significantly exceeding rates in 2022 (0.24%) and 2023 (6.00%) (*χ*^2^ = 500.22, *p* < 0.001). Positivity rates varied significantly by specimen type: sputum exhibited the highest rate at 21.4% (781/3,650), significantly exceeding in oropharyngeal swabs (7.32%, 1,107/15,124) and bronchoalveolar lavage fluid (BALF) (6.59%, 84/1,275) (*χ*^2^ = 674.67, *p* < 0.001). No statistically significant difference was observed between genders from 2022 to 2024 (*χ*^2^ = 0.085, *p* > 0.05). Details are shown in Table 1.

**Table 1:** General characteristics of children with BP infection, 2022–2024.

Variable	2022		2023		2024		*χ* ^2^	*P/P*_trend_
	Number of cases (%)	Positive rate of BP detection (%)	Number of cases (%)	Positive rate of BP detection (%)	Number of cases (%)	Positive rate of BP detection (%)		
BP positive cases	409	14.56	450	3.26	1,113	32.24	2698.35	*P* < 0.001
A2047G mutation number	1 (0.24)	0	27(6)	0.2	477 (42.86)	13.82	500.22	*P* < 0.001
Gender							0.085	*p* = 0.958
Male	225 (55.01)	13.42	245 (54.44)	3.03	615 (55.26)	31.77		
Female	184 (44.99)	16.23	205 (45.56)	3.59	498 (44.74)	32.85		
Type of specimen							674.67	*P* < 0.001
Throat swabs	385 (94.13)	14.35	302 (67.11)	2.79	418 (37.56)	25.63		
Sputum	22 (5.38)	22.22	119 (26.45)	5.59	640 (57.50)	44.98		
BALF	2 (0.49)	7.69	29 (6.44)	3.4	55 (4.94)	13.85		
Other	0(0)	0	0(0)	0	0(0)	0		
Age group								
0–3 M	115 (28.12)	28.33	89 (19.78)	5.36	289 (25.97)	62.42	0.017	*P*_trend_ = 0.897
>3–6 M	95 (23.23)	43.38	47 (10.44)	6.19	130 (11.68)	65.66	25.514	*P*_trend_ < 0.001
>6–12 M	60 (14.67)	11.39	34 (7.56)	1.21	99 (8.89)	23.63	7.965	*P*_trend_ = 0.005
>1–3 Y	22 (5.38)	3.86	28 (6.22)	1.02	61 (5.48)	14.7	0.006	*P*_trend_ = 0.939
>3–6 Y	61 (14.91)	9.05	92 (20.44)	2.91	249 (22.37)	24.95	9.592	*P*_trend_ = 0.002
>6–18 Y	56 (13.69)	13.53	160 (35.56)	6.02	285 (25.61)	29.72	10.436	*P*_trend_ < 0.001
*χ* ^2^	–	281.662	–	190.431	–	393.972	–	–
*P*	–	<0.001	–	<0.001	–	<0.001	–	–

### 3.2 Monthly and annual characteristics of BP infection

**Figure 1:**
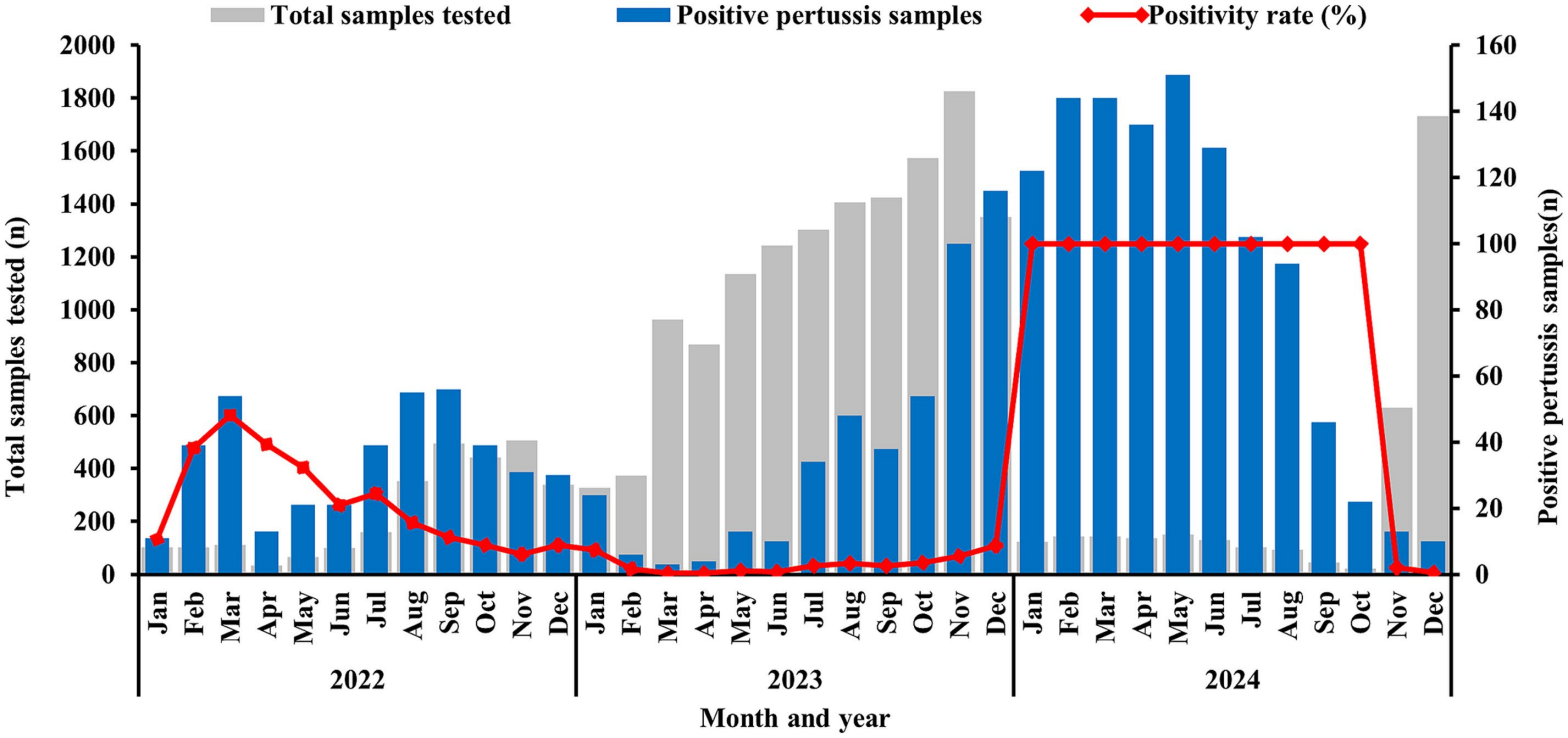
Number and positivity rate of BP-positive specimens, 2022–2024.

Data revealed that BP positive samples were detected throughout all year. In 2022, The first peak BP incidence occurred in March, with a rate of positive specimens of 48.21% (54 positive specimens), followed by a secondary peak of 189 positive specimens from July to October, constituting 46.21% of annual cases. Following this outbreak, the 2023 positivity rate declined sharply to 3.26%, marking a 77.6% reduction from the previous year. However, positive specimens increased monthly from July onward, reaching a peak of 116 positive specimens in December. In 2024, positive specimens surged sharply beginning in January, with a rate of positive specimens of 100% (122 positive specimens) and persisted through October, subsequently declined rapidly, as shown in Figure 1.

### 3.3 Detection rate of children with BP infection in different age groups

**Figure 2:**
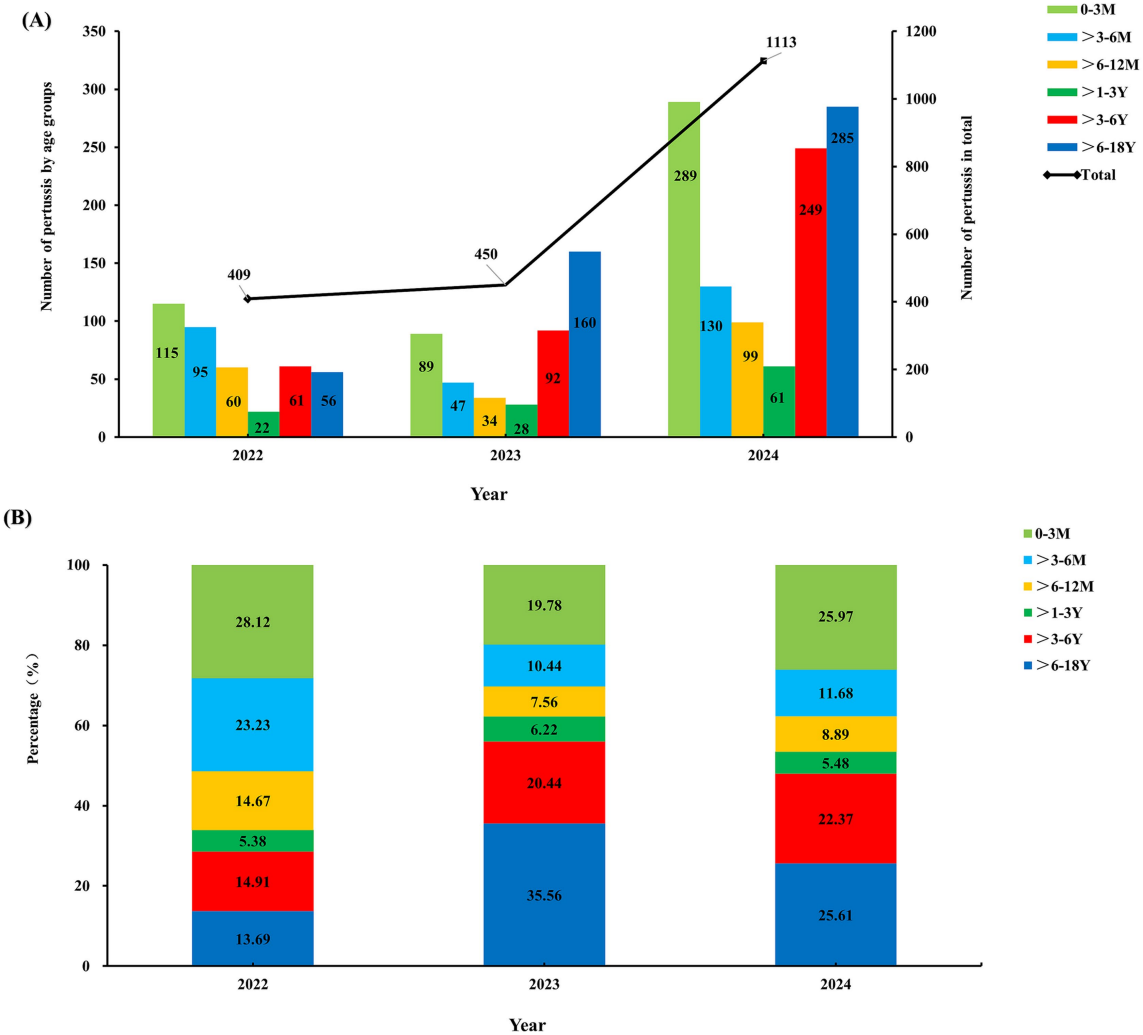
Variation in the count **(A)** and age distribution **(B)** of pertussis, 2022–2024.

Significant differences were observed in both the case counts and age distribution of pertussis from 2022 to 2024, as shown in Figure 2. During the COVID-19 pandemic in 2022, only 409 positive pertussis specimens were identified. In the post-pandemic era from 2023 to 2024, the number of positive specimens increased significantly, particularly in 2024, surging to 1,113 cases (Figure 2A). The age peak of pertussis incidence has shifted. During the pandemic period in 2022, the highest positivity rate occurred among infants <1 year (66.02%), followed by preschool-aged children (>3–6 years) and school-aged children (>6–18 years), accounting for approximately 14.91 and 13.69%, respectively. Toddlers aged >1–3 years represented the smallest proportion, at only 5.38%. However, from 2023 to 2024, the number of cases among school-aged children (>6–18 years) increased significantly, constituting 25.61 to 35.56% of cases, followed by preschool-aged children (>3–6 years), representing approximately 20.44 to 22.37%. The proportion of cases within the >3–6 years and >6–18 years demonstrated statistically significant increasing trends (*P*_trend_ <0.01 and <0.001, respectively). Conversely, the proportion among infants under 1 year decreased, ranging from 37.78 to 46.54%. Toddlers aged >1–3 years consistently remained the population with the lowest proportion, at 6.22% in 2023 and 5.48% in 2024 (Figure 2B).

### 3.4 Annual trends in BP co-detection rates with other pathogens

Among co-detected pathogens, viral pathogens predominated: Human rhinovirus (HRV) accounted for 37.32%, Parainfluenza virus (PIV) for 14.76%, and Adenovirus (ADV) for 13.49%. Bacterial co-infections comprised *Haemophilus influenzae* (HI) (23.23%), *Staphylococcus aureus* (SA) (15.92%), and *Streptococcus pneumoniae* (SP) (23.12%). Among atypical pathogens, *Mycoplasma pneumoniae* (MP) showed a positivity rate of 11.41%. No fungal were co-detected during 2022 to 2024.

**Figure 3:**
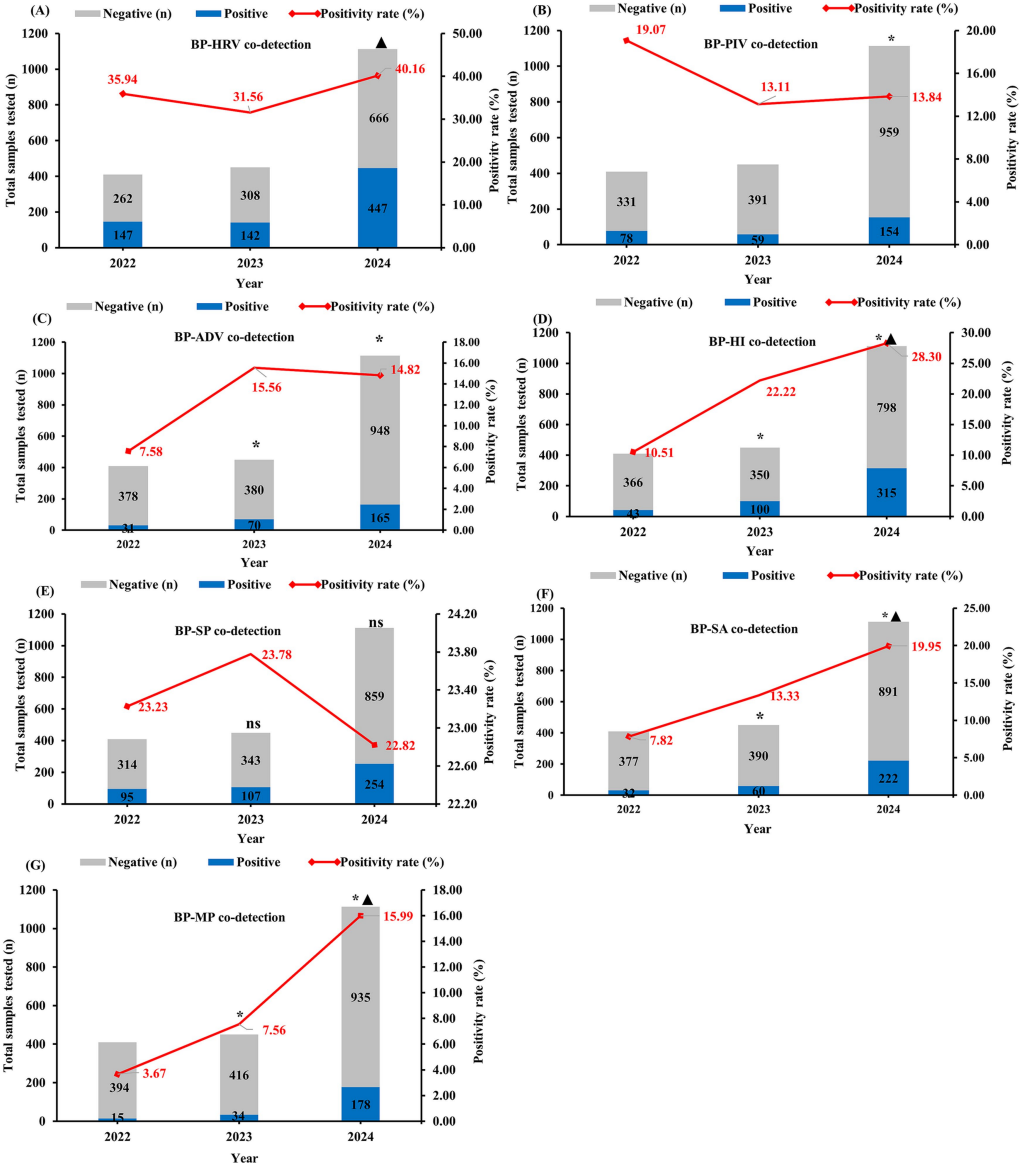
Changes in the number of positive specimens and detection rates of pathogens co-detected with BP, 2022–2024. BP-HRV co-detection rate (*Bordetella pertussis* with Human Rhinovirus), BP-PIV co-detection rate (*Bordetella pertussis* with Parainfluenza Virus), BP-ADV co-detection rate (*Bordetella pertussis* with Adenovirus), BP-HI co-detection rate (*Bordetella pertussis* with *Haemophilus influenzae*), BP-SP co-detection rate (*Bordetella pertussis* with *Streptococcus pneumoniae*), BP-SA co-detection rate (*Bordetella pertussis* with *Staphylococcus aureus*), BP-MP co-detection rate (*Bordetella pertussis* with *Mycoplasma pneumoniae*). * indicates a statistically significant difference (*p* < 0.05) in pathogen co-detection rates compared to 2022 for both 2023 and 2024. ▲ indicates a statistically significant difference (*p* < 0.05) in pathogen co-detection rates between 2023 and 2024.

Figure 3 represents the number of positive cases and detection rates for common pathogens co-detected with BP from 2022 to 2024. Compared to 2022, the co-infection rates of BP with HRV, ADV, HI, SA, and MP significantly increased during 2023–2024 (*χ*^2^ = 4.666, 10.359, 51.926, 35.657, 51.875 respectively; *P*_trend_ < 0.05 for all; Figures 3A,C,D,F,G). Conversely, co-infection with PIV showed a significant decline (*χ*^2^ = 4.863; *P*_trend_ < 0.05; Figure 3B). No significant difference was observed in the co-infection rate with SP (*χ*^2^ = 0.064; *P*_trend_ > 0.05; Figure 3E).

## 4 Discussion

This retrospective study characterized epidemiological shifts in BP infections at the Children’s Hospital Affiliated to Shandong University between 2022 and 2024. Under the backdrop of China’s COVID-19 pandemic initiated late 2019 and full societal reopening in early 2023, data from 2022 reflect pandemic conditions, while 2023–2024 represent the post-pandemic era. Research has found that the epidemiological characteristics of BP infection in children have undergone significant changes: a significant upward trend in BP detection rates, and the proportion of age groups > 3–6 years and above has increased. Seasonal patterns diverged from historical norms: During the pandemic in 2022, the peak in BP positivity rate was concentrated between July and October, similar to the pre-pandemic pattern (7). Post-pandemic in 2023, the peak incidence lasted from July to December, while in 2024, it persisted from January to October. Meanwhile, elevated co-detection rates were observed for BP with HRV, HI, SA, and MP.

This study identified a significantly higher incidence of BP infection in 2024 compared to 2022 and 2023. This shift represents a concrete manifestation of the global resurgence of pertussis and is an inevitable consequence of the pandemic’s impact, attributable to a highly complex interplay of factors. Firstly, waning protection afforded by acellular vaccines over time, along with their effects on infection and transmission, constitutes a key mechanism underlying the pertussis resurgence (21, 22). Secondly, bacterial strain evolution has also driven the resurgence, examples include the increased prevalence of the *ptxP3* allele leading to enhanced toxin expression and mutations in pertactin (*prn*) facilitating vaccine escape (23). Moreover, high macrolide antibiotic resistance has significantly compromised pathogen clearance rates and diminished the capacity to control pathogen transmission (23). Within this context, the implementation of Non-Pharmaceutical Interventions (NPIs) during the COVID-19 pandemic reduced population-level pathogen exposure. This attenuation of naturally acquired herd immunity (24), coupled with partial vaccination delays creating an “immunity debt” (8), resulted in an expanded pool of susceptible individuals. Consequently, a resurgence in the activity of respiratory pathogens occurred following the relaxation of NPIs. Furthermore, social distancing measures during the COVID-19 delay or avoid medical consultations. Following the relaxation of NPIs, the restoration of routine medical services resulted in a surge of medical demand. This change in healthcare-seeking behavior partially explains the resurgence of pertussis (25). Concurrently, the widespread use of PCR testing during the COVID-19 increased public acceptance of this method. Especially the expanded use of multiplex PCR assays detecting BP has improved case detection and reporting efficiency. Additionally, heightened disease awareness among doctors during the pandemic also contributed to the increase in the number of tests and case detection rates (25). After the pandemic, clinicians and public health agencies have increased awareness of the resurgence of respiratory pathogens, which has led to strengthened pertussis surveillance, shifting from passive to active monitoring (12). All of the above are important factors contributing to the observed resurgence trend of pertussis. In the case where there were no changes in the detection platform, sampled population, and testing coverage throughout the period, pertussis detection rates in 2023 decreased significantly compared to adjacent years. This may be related to the adjustment of COVID-19 policies. After the full societal reopening in early 2023, a surge in respiratory pathogens substantially increased testing demand and total specimen volume. Although positive pertussis samples increased after July 2023, this occurred against a background of expanded testing volume, resulting in a relatively low positivity rate.

Notably, a sharp decrease in pertussis cases occurred after October 2024. Possible reasons include the following: first, the potential establishment of herd immunity. Pertussis transmission depends on the accumulation of susceptible individuals. Persistent high incidence of pertussis from July 2023 to October 2024 may have led to the acquisition of immunity through natural infection, reducing susceptibility and interrupting transmission chains. Research by Lavine et al. (24) demonstrated that the enhanced immunity induced by natural infection can significantly affect the epidemic dynamics of pertussis and reduce the subsequent transmission risk. Second, seasonal influences. Although NPIs disrupted traditional seasonality, climate factors (temperature/humidity variations) remain relevant. Dan et al. (7) identified that the peak pertussis incidence in spring and summer, respiratory diseases caused by influenza and RSV in winter indirectly reduced pertussis circulation (13). Third, enhanced control measures. Responding to the 2023–2024 surge, in June 2024, Chinese Center for Disease Control and Prevention (CDC) and the National Health Commission jointly issued the “Guidelines for diagnosis and management and prevention of pertussis of China (2024 edition)” (26). These interventions focused on key populations and institutions through strengthened vaccination and health education, enhancing respiratory hygiene protocols with case isolation, and implementation of multi-channel surveillance systems. Consequently, transmission rates of pertussis were significantly reduced, contributing to case reduction. Given the sustained efficacy of these measures, pertussis is expected to remain at sporadic levels over the next 1–2 years. Concurrently, this study observed the A2047G mutation showed an annual rise in detection, nearing 50% in 2024, with the potential to exacerbate pertussis transmission. Given the persistent challenges of rising pertussis incidence, escalating antibiotic resistance, and enhanced bacterial virulence, there is an urgent warrant to strengthen surveillance specifically targeting drug-resistant BP.

Meanwhile, this study observed a significant increase in the proportion of patients aged >3–6 years and older from 2022 to 2024, suggesting a shift in BP infections toward older children. This finding aligns with research by Hu et al. (27). This epidemiological transition is primarily attributed to waning vaccine-induced immunity over time and insufficient booster vaccination. While complete vaccination records were unavailable in this study, a Korean study found 92.4% of pertussis cases occurred among fully vaccinated school-aged children (28), suggesting waning DTaP immunity may be a key factor. Previous literature indicates pertussis immunity significantly decline 4 years after vaccination (29). Moreover, booster vaccination coverage was insufficient, with rates of only 19.0% among 5-year-olds and 15.6% among 6-year-olds (30). These findings suggest that the waning vaccine immunity and inadequate booster coverage in the rising pertussis incidence among Chinese preschool and school-aged children. Notably, early infant primary immunization, when coupled with preschool and adolescent booster doses, has been demonstrated to effectively reduce morbidity and mortality in young infants (31, 32). China has recently updated the DTaP (Diphtheria, Tetanus, acellular Pertussis) vaccination schedule to include doses at 2, 4, 6, and 18 months, with a booster at 6 years of age. This update reflects heightened national focus on protecting high-risk populations. Secondly, significant evolution has occurred in the predominant genotypes of macrolide-resistant *B. pertussis* (MRBP). Prior to 2020, MRBP strains were almost exclusively the low-virulence ptxP1 genotype (33). Although the high-virulence ptxP3 genotype was first identified in 2002, it remained erythromycin-susceptible for an extended period (34). It was not until 2022 that the Wu X team first reported ptxP3-MRBP strains (35). Recent research conducted amidst the post-COVID pertussis surge suggests that the emergence and spread of the highly virulent ptxP3-MRBP-MT28 strain may be a key driver behind the surge in domestic cases, the shift in age distribution, and vaccine escape phenomena (36). As virulence genotyping was not performed in this study, assessment of *ptxP3* prevalence was precluded. To resolve this problem, future studies should implement whole-genome sequencing to achieve a mechanistic analysis of the driving factors behind the resurgence of pertussis. These older infected children potentially serve as significant reservoirs of infection for unvaccinated infants (37). Critically, despite a rising pertussis incidence among school-aged children from 2022 to 2024, the incidence rate in infants <3 months remained consistently high (28.12% in 2022, 19.78% in 2023, and 25.97% in 2024). For this pre-vaccination population, maternal Tdap vaccination during gestational weeks 27–36 is the most effective strategy to prevent severe pertussis (38). Importantly, as a complementary measure, China will advance the first DTaP dose from 3 months to 2 months of age from January 2025, enhancing early protection for this vulnerable population. Previous studies by our team have demonstrated recent or active pertussis infection in 0.8% of hospitalized pneumonia cases and 10% of asthma cases, often presenting without typical symptoms (39). Among children with prolonged cough (>2 weeks), the pertussis diagnosis rate reached 21.4%, predominantly in those aged 7–14 years (40). Furthermore, our multi-center study within the province revealed that 26.22% of children with asthma had concurrent pertussis infection, with a particularly high prevalence in those ≥6 years old (41). Collectively, these results indicate that school-aged children have emerged as a crucial high-risk group for pertussis. Moreover, the infection frequently presents atypically, leading to potential misdiagnosis. Therefore, enhancing clinical recognition capabilities among healthcare professionals is paramount to interrupting disease transmission.

Mixed infections have garnered attention due to their potential to exacerbate pertussis severity (42). Studies indicate that 76.7% of BP infections involve co-infection pathogens (43). Although respiratory syncytial virus (RSV), parainfluenza virus 3 (PIV-3), and ADV are frequently reported as common co-infecting agents in both domestic and international research (44–46), substantial heterogeneity exists regarding their prevalence profiles and specific types. For instance, Scutari et al. identified HRV, human metapneumovirus (HMPV), and PIV-3 as closely associated with BP infection (43); Muloiwa et al. observed a doubled risk of PIV infection among BP patients (47); conversely, Gan and Wu (48) found Gram-negative bacteria and PIV-3 predominating in unvaccinated infants. In the present study, we identified HRV as the predominant co-detected pathogen, followed by bacteria co-detections—primarily HI and SP. Notably, the high prevalence of HI and SP validates the WHO’s 2008 *Global Action Plan for Pneumonia Prevention and Control*, which designates vaccination against these pathogens as primary prevention. Consistent with this framework, *Haemophilus influenzae* type b (Hib) vaccine was introduced and conducted research in 1996 in our country (49), implementing a tiered schedule: 3-dose primary series (≥28-day intervals) for infants aged 2–5 months plus 18-month booster; 2-dose primary series for 6–11-month infants with booster; single dose for children aged 1–5 years. For pneumococcal vaccines, 13-Valent Pneumococcal Conjugate Vaccine (PCV13) (6 weeks-5 years) follows a 2/4/6-month primary series with 12–15-month booster, while catch-up schedules differ by age: 2 + 1 doses for 7–11-month infants (2-month interval, booster ≥12 month), 2 doses for 12–23-month toddlers, and single dose for 2–5-year children. Neither vaccine is currently included in China’s national immunization program, more research is needed to bridge this policy gap. Importantly, our study did not integrate clinical symptoms, thus the co-detection rate in the study should not be equated with co-infection rate. This difference is particularly crucial for pathogens like SP, which are known to have nasopharyngeal colonization dynamics. Consequently, careful clinical assessment and discrimination are essential during diagnosis and management to accurately identify the causative pathogens responsible for the infection.

This study revealed that during the post-pandemic era (2023–2024), the co-detection rates of HRV, ADV, HI, and SA with BP were significantly higher compared to the pandemic year 2022. This trend aligns with predictions made by Shi et al. (50). The increase in HRV and ADV infections may be linked to their high asymptomatic carriage rates, unique transmission mechanisms, and the environmental stability characteristic of non-enveloped viruses (resistance to drying, acid, and alcohol) (51, 52). Conversely, the rise in bacterial infections likely stems from the removal of transmission restrictions following the discontinuation of NPIs (53), coupled with school re-openings and declining adherence to protective measures among children (50). Notably, a declining trend in PIV infection rates was observed, contrasting with reports by Wang et al. (54). This divergence suggests that NPIs may have altered population pathogen exposure profiles and the complexity of respiratory coinfections in children. Furthermore, a significantly elevated detection rate of MP was found in this study, consistent with the conclusions of Jiang et al. (55–57). This increase is potentially attributable to the accumulation of susceptible individuals due to suppressed transmission during NPIs, potential antigenic variation (58), and attenuated humoral immunity (59). Critically, we emphasize the high degree of overlap observed between MP, a pathogen commonly associated with preschool and school-age children, and BP infections, which are more typical in younger children. This finding underscores the rapidly evolving landscape of pediatric respiratory infection etiology, presenting novel challenges for future infection prevention and control strategies. In summary, the dynamic evolution of BP mixed infections is shaped by the complex interplay of multiple factors. The epidemic characteristics of the post-pandemic era are collectively molded by the specific properties of pathogens (e.g., environmental stability, transmission mechanisms), population attributes (age and immune background), geographical variations, and the stringency and duration of control measures (NPIs) implemented during different periods. Consequently, achieving more precise, effective, and rapid identification and diagnosis holds significant practical and societal importance.

In this study, we employed T-NGS technology for pathogen detection. As an advanced technology, it can be categorized into multiplex PCR-based T-NGS (mp-T-NGS) and hybrid capture-based T-NGS (hc-T-NGS) according to its technical route. Literature shows that compared with the composite reference standard, the sensitivity of mp-T-NGS and hc-T-NGS was 86.5 and 87.3%, respectively, while the specificity reached 90.0 and 88.0%, respectively (18). For common pathogens, the detection rates of mp-T-NGS and hc-T-NGS were 84.3 and 89.5%, respectively, significantly higher than the detection rate of 88.5% of traditional microbial detection methods (including culture, qPCR, serological testing, [1,3]-β-D-glucan, and galactomannan assays) (18). When comparing the diagnostic value of T-NGS and PCR for pertussis, the receiver operating characteristic (ROC) curves shows that T-NGS achieved a significantly higher area under the curve (AUC) of 0.812 than PCR (0.787) and the sensitivity is also significantly higher than that of the PCR method (90% vs. 85.2%), suggesting superior diagnostic value for pertussis (16). Under the trend of rising macrolide antibiotic resistance, T-NGS can identify pathogen subtypes and detect resistance gene mutation sites, which are superior to those of conventional PCR methods. Although T-NGS has high sensitivity in pathogen detection, its performance can be affected by enrichment bias and primer/probe coverage. These limitations can be effectively fixed by improving the specificity of primer/probe design, optimizing experimental protocols, standardizing sequencing depth in data analysis, utilizing bioinformatics tools to improve primer coverage efficiency, and conducting comprehensive performance evaluations through both dry laboratory and wet laboratory validation (17). In the future, the implementation of NGS will require standardized framework to translate the technological promise into clinical precision.

This study has several limitations. (1) As a single-center investigation, its findings primarily reflect regional epidemiological characteristics of pertussis. (2) Constrained by the retrospective study design and data access, our study lacks integration of clinical symptom and outcome data (such as symptom duration, hospitalization outcomes), could not explore potential associations between molecular epidemiology and disease severity. (3) Pathogen virulence genotyping was not performed, limiting mechanistic insights into resurgence. Future multi-center cohort studies will incorporate both pathogen whole-genome sequencing and host genotyping alongside clinical data to more comprehensively assess the long-term impact of epidemiological shifts on disease burden.

The research method aligns well with the study objectives. The selection of T-NGS technology, application of statistical methods, and the quality control measures are appropriate, establishing a solid foundation for the reliability of the results. The results integrate global mechanisms behind the resurgence of pertussis (such as waning vaccine immunity and the impact of NPIs) and logically link them to China’s prevention and control policies. However, the interpretation inadequately addresses the potential for detection bias associated with advances in T-NGS, where co-detection may overestimate BP infection, and virulence gene typing data were not included. Although drug resistance gene detection was conducted during T-NGS analysis, the absence of specific minimum inhibitory concentration (MIC) results limits a deeper interpretation of the mechanisms driving the pertussis resurgence.

Our study also has several strengths. First, it employed clear inclusion criteria and enrolled a substantial sample of 20,059 cases at a regional medical center (Jinan, China), covering all age groups from 0 to 18 years. The data includes outpatient, emergency, and inpatient cases, covering the key population from multiple dimensions. In addition, the study period spanned the COVID-19 prevention and control period (2022) to the post-pandemic era (2023–2024), fully capturing the transition period of the pandemic and directly reflecting the changes in the epidemiological characteristics of pertussis after the lifting of NPIs. Third, advanced technology of T-NGS was uniformly applied throughout the research period to avoid methodological heterogeneity. Strict quality control procedures were implemented to ensure the reliability of the results. Finally, the molecular epidemiological data were translated into empirical evidence that can directly guide clinical practice and public health interventions, providing key regional evidence for global pertussis prevention and control.

## 5 Conclusion

This large-scale retrospective study leveraging a province with a substantial population base (100 million) and historically high pertussis reporting rates and incorporating over 20,000 specimens, has revealed significant alterations in the epidemiological characteristics of pediatric BP infections during the post-pandemic era, demonstrating increased incidence, non-seasonal surges, a rising proportion of cases among school-aged children, the year-on-year escalation of macrolide resistance mutations (notably A2047G), and a higher prevalence of co-detections; these findings underscore the necessity to enhance surveillance specifically targeting drug-resistant BP, strengthen vaccination strategies (particularly focusing on school-aged children in close contact with infants), improve clinical diagnostic capabilities, and implement targeted public health interventions to interrupt transmission, thereby providing critical evidence to inform precision surveillance systems and refine clinical diagnosis and management practices.

## Data Availability

The datasets presented in this study can be found in online repositories. The names of the repository/repositories and accession number(s) can be found in the article/Supplementary material.

## Footnotes

^1^ https://github.com/brwnj/bcl2fastq

^2^ https://www.bioinformatics.babraham.ac.uk/projects/fastqc/

^3^ https://ftp.ncbi.nlm.nih.gov/genomes/genbank/
