# Supplementary material for: Molecular epidemiological characteristics of pertussis in a pediatric hospital in Jinan City, China, 2022 to 2024
Source: Front Public Health. 2025 Sep 2;13:1657953. doi: 10.3389/fpubh.2025.1657953 (PMC12436086; doi:10.3389/fpubh.2025.1657953)
Supplement: Supplementary file 1 [file Table_1.DOCX]

| Changes in the number of positive specimens and detection rates of pathogens co-detected with BP, 2022–2024. | | | | | | | | | | | |
| --- | --- | --- | --- | --- | --- | --- | --- | --- | --- | --- | --- |
| Human rhinovirus | 2022 | | | 2023 | | | 2024 | | | χ² | *P* _trend_ |
| Month | *Number of specimens*  *in total* | *Number of positive specimens* | *Positivity rate (%)* | Number of specimens  in total | Number of positive specimens | Positivity rate (%) | Number of specimens  in total | Number of positive specimens | Positivity rate (%) | 4.666 | 0.032 |
| Jan | *11* | *1* | *9.09* | 24 | 2 | 8.33 | 122 | 43 | 35.25 |  |  |
| Feb | *39* | *13* | *33.33* | 6 | 1 | 16.67 | 144 | 43 | 29.86 |  |  |
| Mar | *54* | *19* | *35.19* | 3 | 2 | 66.67 | 144 | 53 | 36.81 |  |  |
| Apr | *13* | *3* | *23.08* | 4 | 2 | 50.00 | 136 | 75 | 55.15 |  |  |
| May | *21* | *10* | *47.62* | 13 | 6 | 46.15 | 151 | 76 | 50.33 |  |  |
| Jun | *21* | *11* | *52.38* | 10 | 5 | 50.00 | 129 | 49 | 37.98 |  |  |
| Jul | *39* | *19* | *48.72* | 34 | 9 | 26.47 | 102 | 27 | 26.47 |  |  |
| Aug | *55* | *17* | *30.91* | 48 | 15 | 31.25 | 94 | 31 | 32.98 |  |  |
| Sep | *56* | *16* | *28.57* | 38 | 17 | 44.74 | 46 | 23 | 50.00 |  |  |
| Oct | *39* | *12* | *30.77* | 54 | 29 | 53.70 | 22 | 15 | 68.18 |  |  |
| Nov | *31* | *20* | *64.52* | 100 | 33 | 33.00 | 13 | 7 | 53.85 |  |  |
| Dec | *30* | *6* | *20.00* | 116 | 20 | 17.24 | 10 | 5 | 50.00 |  |  |
| Parainfluenza virus | *2022* | | | 2023 | | | 2024 | | | χ² | *P* _trend_ |
| Month | *Number of specimens*  *in total* | *Number of positive specimens* | *Positivity rate (%)* | Number of specimens  in total | Number of positive specimens | Positivity rate (%) | Number of specimens  in total | Number of positive specimens | Positivity rate (%) | 4.863 | 0.03 |
| Jan | *11* | *6* | *54.55* | 24 | 0 | 0.00 | 122 | 4 | 3.28 |  |  |
| Feb | *39* | *7* | *17.95* | 6 | 0 | 0.00 | 144 | 3 | 2.08 |  |  |
| Mar | *54* | *7* | *12.96* | 3 | 0 | 0.00 | 144 | 14 | 9.72 |  |  |
| Apr | *13* | *2* | *15.38* | 4 | 0 | 0.00 | 136 | 13 | 9.56 |  |  |
| May | *21* | *1* | *4.76* | 13 | 0 | 0.00 | 151 | 36 | 23.84 |  |  |
| Jun | *21* | *3* | *14.29* | 10 | 2 | 20.00 | 129 | 29 | 22.48 |  |  |
| Jul | *39* | *13* | *33.33* | 34 | 11 | 32.35 | 102 | 26 | 25.49 |  |  |
| Aug | *55* | *19* | *34.55* | 48 | 14 | 29.17 | 94 | 15 | 15.96 |  |  |
| Sep | *56* | *12* | *21.43* | 38 | 11 | 28.95 | 46 | 9 | 19.57 |  |  |
| Oct | *39* | *4* | *10.26* | 54 | 9 | 16.67 | 22 | 3 | 13.64 |  |  |
| Nov | *31* | *2* | *6.45* | 100 | 9 | 9.00 | 13 | 1 | 7.69 |  |  |
| Dec | *30* | *2* | *6.67* | 116 | 3 | 2.59 | 10 | 1 | 10.00 |  |  |
| Adenovirus | *2022* | | | 2023 | | | 2024 | | | χ² | *P* _trend_ |
| Month | *Number of specimens*  *in total* | *Number of positive specimens* | *Positivity rate (%)* | Number of specimens  in total | Number of positive specimens | Positivity rate (%) | Number of specimens  in total | Number of positive specimens | Positivity rate (%) | 10.359 | ＜0.001 |
| Jan | *11* | *0* | *0.00* | 24 | 1 | 4.17 | 122 | 23 | 18.85 |  |  |
| Feb | *39* | *5* | *12.82* | 6 | 1 | 16.67 | 144 | 29 | 20.14 |  |  |
| Mar | *54* | *6* | *11.11* | 3 | 0 | 0.00 | 144 | 20 | 13.89 |  |  |
| Apr | *13* | *3* | *23.08* | 4 | 0 | 0.00 | 136 | 18 | 13.24 |  |  |
| May | *21* | *1* | *4.76* | 13 | 0 | 0.00 | 151 | 16 | 10.60 |  |  |
| Jun | *21* | *1* | *4.76* | 10 | 3 | 30.00 | 129 | 20 | 15.50 |  |  |
| Jul | *39* | *3* | *7.69* | 34 | 4 | 11.76 | 102 | 15 | 14.71 |  |  |
| Aug | *55* | *5* | *9.09* | 48 | 8 | 16.67 | 94 | 13 | 13.83 |  |  |
| Sep | *56* | *3* | *5.36* | 38 | 3 | 7.89 | 46 | 6 | 13.04 |  |  |
| Oct | *39* | *0* | *0.00* | 54 | 14 | 25.93 | 22 | 4 | 18.18 |  |  |
| Nov | *31* | *2* | *6.45* | 100 | 16 | 16.00 | 13 | 0 | 0.00 |  |  |
| Dec | *30* | *2* | *6.67* | 116 | 20 | 17.24 | 10 | 1 | 10.00 |  |  |
| *Haemophilus influenzae* | *2022* | | | 2023 | | | 2024 | | | χ² | *P* _trend_ |
| Month | *Number of specimens*  *in total* | *Number of positive specimens* | *Positivity rate (%)* | Number of specimens  in total | Number of positive specimens | Positivity rate (%) | Number of specimens  in total | Number of positive specimens | Positivity rate (%) | 51.926 | ＜0.001 |
| Jan | *11* | *2* | *18.18* | 24 | 3 | 12.50 | 122 | 31 | 25.41 |  |  |
| Feb | *39* | *6* | *15.38* | 6 | 0 | 0.00 | 144 | 59 | 40.97 |  |  |
| Mar | *54* | *8* | *14.81* | 3 | 0 | 0.00 | 144 | 45 | 31.25 |  |  |
| Apr | *13* | *1* | *7.69* | 4 | 0 | 0.00 | 136 | 41 | 30.15 |  |  |
| May | *21* | *2* | *9.52* | 13 | 1 | 7.69 | 151 | 34 | 22.52 |  |  |
| Jun | *21* | *2* | *9.52* | 10 | 0 | 0.00 | 129 | 32 | 24.81 |  |  |
| Jul | *39* | *4* | *10.26* | 34 | 5 | 14.71 | 102 | 24 | 23.53 |  |  |
| Aug | *55* | *3* | *5.45* | 48 | 15 | 31.25 | 94 | 18 | 19.15 |  |  |
| Sep | *56* | *1* | *1.79* | 38 | 8 | 21.05 | 46 | 17 | 36.96 |  |  |
| Oct | *39* | *4* | *10.26* | 54 | 18 | 33.33 | 22 | 7 | 31.82 |  |  |
| Nov | *31* | *8* | *25.81* | 100 | 16 | 16.00 | 13 | 4 | 30.77 |  |  |
| Dec | *30* | *2* | *6.67* | 116 | 33 | 28.45 | 10 | 3 | 30.00 |  |  |
| *Streptococcus pneumoniae* | *2022* | | | 2023 | | | 2024 | | | χ² | *P* _trend_ |
| Month | *Number of specimens*  *in total* | *Number of positive specimens* | *Positivity rate (%)* | Number of specimens  in total | Number of positive specimens | Positivity rate (%) | Number of specimens  in total | Number of positive specimens | Positivity rate (%) | 0.064 | 0.816 |
| Jan | *11* | *1* | *9.09* | 24 | 4 | 16.67 | 122 | 28 | 22.95 |  |  |
| Feb | *39* | *8* | *20.51* | 6 | 2 | 33.33 | 144 | 25 | 17.36 |  |  |
| Mar | *54* | *10* | *18.52* | 3 | 1 | 33.33 | 144 | 32 | 22.22 |  |  |
| Apr | *13* | *3* | *23.08* | 4 | 1 | 25.00 | 136 | 34 | 25.00 |  |  |
| May | *21* | *5* | *23.81* | 13 | 4 | 30.77 | 151 | 31 | 20.53 |  |  |
| Jun | *21* | *7* | *33.33* | 10 | 7 | 70.00 | 129 | 27 | 20.93 |  |  |
| Jul | *39* | *12* | *30.77* | 34 | 7 | 20.59 | 102 | 20 | 19.61 |  |  |
| Aug | *55* | *14* | *25.45* | 48 | 7 | 14.58 | 94 | 25 | 26.60 |  |  |
| Sep | *56* | *10* | *17.86* | 38 | 8 | 21.05 | 46 | 16 | 34.78 |  |  |
| Oct | *39* | *15* | *38.46* | 54 | 15 | 27.78 | 22 | 6 | 27.27 |  |  |
| Nov | *31* | *7* | *22.58* | 100 | 26 | 26.00 | 13 | 6 | 46.15 |  |  |
| Dec | *30* | *3* | *10.00* | 116 | 25 | 21.55 | 10 | 4 | 40.00 |  |  |
| *Staphylococcus aureus* | *2022* | | | 2023 | | | 2024 | | | χ² | *P* _trend_ |
| Month | *Number of specimens*  *in total* | *Number of positive specimens* | *Positivity rate (%)* | Number of specimens  in total | Number of positive specimens | Positivity rate (%) | Number of specimens  in total | Number of positive specimens | Positivity rate (%) | 35.657 | ＜0.001 |
| Jan | *11* | *0* | *0.00* | 24 | 5 | 20.83 | 122 | 24 | 19.67 |  |  |
| Feb | *39* | *3* | *7.69* | 6 | 1 | 16.67 | 144 | 26 | 18.06 |  |  |
| Mar | *54* | *6* | *11.11* | 3 | 1 | 33.33 | 144 | 34 | 23.61 |  |  |
| Apr | *13* | *2* | *15.38* | 4 | 0 | 0.00 | 136 | 23 | 16.91 |  |  |
| May | *21* | *1* | *4.76* | 13 | 0 | 0.00 | 151 | 33 | 21.85 |  |  |
| Jun | *21* | *3* | *14.29* | 10 | 1 | 10.00 | 129 | 23 | 17.83 |  |  |
| Jul | *39* | *3* | *7.69* | 34 | 2 | 5.88 | 102 | 23 | 22.55 |  |  |
| Aug | *55* | *3* | *5.45* | 48 | 4 | 8.33 | 94 | 14 | 14.89 |  |  |
| Sep | *56* | *3* | *5.36* | 38 | 7 | 18.42 | 46 | 10 | 21.74 |  |  |
| Oct | *39* | *2* | *5.13* | 54 | 4 | 7.41 | 22 | 7 | 31.82 |  |  |
| Nov | *31* | *3* | *9.68* | 100 | 13 | 13.00 | 13 | 3 | 23.08 |  |  |
| Dec | *30* | *3* | *10.00* | 116 | 22 | 18.97 | 10 | 2 | 20.00 |  |  |
| *Mycoplasma pneumoniae* | *2022* | | | 2023 | | | 2024 | | | χ² | *P* _trend_ |
| Month | *Number of specimens*  *in total* | *Number of positive specimens* | *Positivity rate (%)* | Number of specimens  in total | Number of positive specimens | Positivity rate (%) | Number of specimens  in total | Number of positive specimens | Positivity rate (%) | 53.562 | ＜0.001 |
| Jan | *11* | *0* | *0.00* | 24 | 1 | 4.17 | 122 | 20 | 16.39 |  |  |
| Feb | *39* | *1* | *2.56* | 6 | 0 | 0.00 | 144 | 17 | 11.81 |  |  |
| Mar | *54* | *6* | *11.11* | 3 | 0 | 0.00 | 144 | 9 | 6.25 |  |  |
| Apr | *13* | *1* | *7.69* | 4 | 0 | 0.00 | 136 | 19 | 13.97 |  |  |
| May | *21* | *0* | *0.00* | 13 | 0 | 0.00 | 151 | 12 | 7.95 |  |  |
| Jun | *21* | *1* | *4.76* | 10 | 0 | 0.00 | 129 | 16 | 12.40 |  |  |
| Jul | *39* | *3* | *7.69* | 34 | 1 | 2.94 | 102 | 28 | 27.45 |  |  |
| Aug | *55* | *1* | *1.82* | 48 | 0 | 0.00 | 94 | 30 | 31.91 |  |  |
| Sep | *56* | *0* | *0.00* | 38 | 0 | 0.00 | 46 | 10 | 21.74 |  |  |
| Oct | *39* | *1* | *2.56* | 54 | 5 | 9.26 | 22 | 8 | 36.36 |  |  |
| Nov | *31* | *0* | *0.00* | 100 | 13 | 13.00 | 13 | 4 | 30.77 |  |  |
| Dec | *30* | *1* | *3.33* | 116 | 14 | 12.07 | 10 | 5 | 50.00 |  |  |
